# Correction: ICAM-1 controls development and function of ILC2

**DOI:** 10.1084/jem.2017235906182025c

**Published:** 2025-06-25

**Authors:** Ai-Hua Lei, Qiang Xiao, Gao-Yu Liu, Kun Shi, Qiong Yang, Xing Li, Yu-Feng Liu, Hai-Kun Wang, Wei-Ping Cai, Yu-Juan Guan, Dmitry I. Gabrilovich, Jie Zhou

Vol. 215, No. 8 | https://doi.org/10.1084/jem.20172359 | July 26, 2018

The authors regret that, in their original article, the representative flow cytometric image of the *ICAM-1*^*−/−*^ group in Fig. 5 E (not included in the statistical data) was mistakenly taken from a different group during figure preparation. This error does not affect the conclusions of the study, and the figure legend remains unchanged. The original and corrected figures are shown here. The error appears in print and in PDFs downloaded before June 18, 2025.

**Figure:**
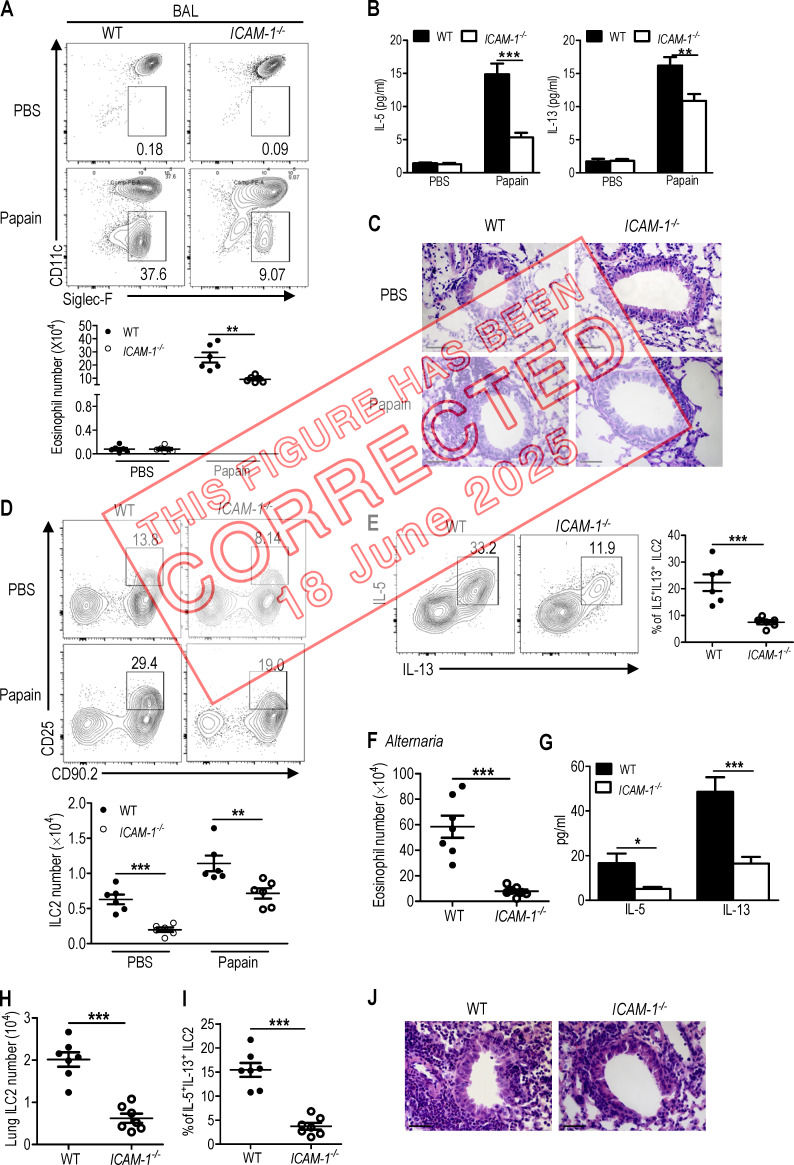


**Figure 5.**
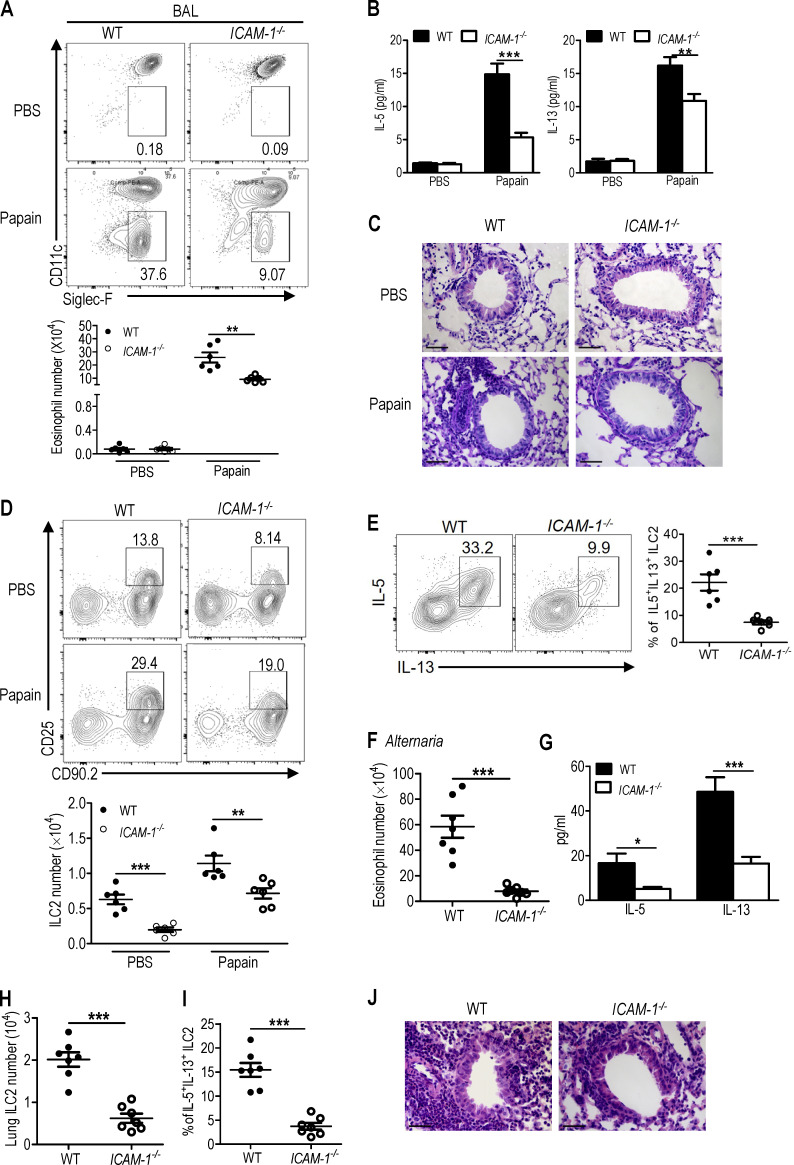
**ICAM-1 deficiency attenuates papain and *A. alternata*–induced lung inflammation. (A–E)** WT and *ICAM-1*^*−/−*^ mice were intranasally administered with papain or PBS for 5 d (*n* = 6 for each group). Mice were sacrificed 24 h after the last treatment. **(A)** The frequencies and number of eosinophils in BAL were evaluated by flow cytometry. **(B)** The amount of IL-5 and IL-13 in BAL was measured by ELISA. **(C)** Representative lung histology by H&E staining (bars, 100 µm). **(D)** The frequencies and number of lung ILC2s were determined by flow cytometry. **(E)** Flow cytometric analysis of frequencies of IL-5^+^IL-13^+^ in lung ILC2s after cell stimulation cocktail treatment for 4 h. Papain treated groups were analyzed. **(F–J)** WT and *ICAM-1*^*−/−*^ mice were intranasally challenged with extract of *A. alternata* for 4 d (*n* = 7). Mice were sacrificed 24 h after the last challenge. **(F)** The number of eosinophils in BAL was evaluated by flow cytometry. **(G)** The amount of IL-5 and IL-13 in BAL was measured by ELISA. **(H)** Total number of ILC2 in lung after *A. alternata* treatment. **(I)** The frequencies of IL-5^+^IL-13^+^ in lung ILC2s after cell stimulation cocktail treatment for 4 h. **(J)** Representative H&E staining of lung sections in *A. alternata*–treated groups (bars, 100 µm). Data are representative of two independent experiments. Error bars show mean ± SEM; **, P < 0.01; ***, P < 0.001 by unpaired Student's *t* test. Numbers within flow plots indicate the percentages of cells gated.

